# Tetanic Symptoms from the Use of Iodide of Potassium

**Published:** 1853-05

**Authors:** D. P. Phillips

**Affiliations:** Passed Assistant Surgeon, U. S. N.


*Tetanic Symptoms from the use of Iodide of Potassium.* By D. P. Phillips, M. D., Passed Assistant Surgeon, U. S. N. (Communicated by Prof. Dunglison.)

A case of some singularity having occurred under my own observation, and thinking that it might not be devoid of interest to you, I have concluded briefly to give its history.

Whilst Acting Surgeon of the U. S. Ship Massachusetts, a fireman, named J. White, was admitted upon my sick list with rheumatism. I ordered the administration of iodide of potassium, grs. viii. ter in die, to be taken before meals in a spoonful of water. Soon after commencing with the remedy (probably the second day) he complained of some uneasiness and stiffness in the jaws; but supposing it to be some trivial affair, I paid but little attention to it. On the next day the difficulty had increased, and I directed frictions with some stimulating liniment; but when I saw him the day after, the jaws were immoveable. Upon careful inquiry, I ascertained that ever since he had been using the iodide he had experienced a burning and uneasy sensation in the œsophagus and stomach. Upon learning this I discontinued the medicine, and ordered counter-irritation over the stomach. In a few days the tetanic symptoms entirely disappeared, and the iodide of potassium was renewed, but diluted in a tumbler half full of water, and given *after* each meal. The patient entirely recovered from rheumatism, and had no return of the trismus. I attributed the unusual symptoms entirely to the use of iodide of potassium in too concentrated a form.

